# Ten weeks of 100% orange juice consumption had a marginal effect on women's skin health compared to a low-flavanone orange-flavored control beverage: a pilot randomized trial

**DOI:** 10.3389/fnut.2025.1648394

**Published:** 2025-09-03

**Authors:** Wenyi Fu, Liwei Gu

**Affiliations:** Department of Food Science and Human Nutrition, Institute of Food and Agricultural Sciences, University of Florida, Gainesville, FL, United States

**Keywords:** orange juice, UVB-induced erythema, skin health, oxidative stress, inflammation

## Abstract

Human skin health deteriorates after age 40, particularly due to ultraviolet (UV) radiation, a major extrinsic factor contributing to photoaging. This study investigated whether daily consumption of 100% orange juice could reduce UVB-induced erythema, improve skin health, and lower biomarkers of oxidative stress and inflammation. A randomized, single-blinded, crossover trial was conducted in 24 healthy women aged 40–65. Participants consumed 12 oz of 100% orange juice or an orange-flavored control beverage daily for 10 weeks, followed by a 28-day washout before switching beverages for another 10 weeks. Skin assessments and blood sample collection were conducted at baseline, week 5, and week 10. Orange juice offers significantly higher daily flavanone content (81.46 mg/day) than the control beverage (29.60 mg/day). Ten weeks of orange juice consumption significantly reduced forearm wrinkles and showed a trend toward reduced skin roughness. However, no significant improvements were observed in UVB-induced erythema and other skin health parameters, including transepidermal water loss, skin hydration, or elasticity. Furthermore, blood levels of matrix metalloproteinase-9, advanced glycation end products, superoxide dismutase, glutathione peroxidase, IL-6, TNF-α, and high-sensitivity C-reactive protein remained unchanged. In conclusion, daily consumption of orange juice for 10 weeks had a marginal effect on skin health.

## 1 Introduction

The skin is the largest organ of the human body. It performs pivotal physiological and protective functions, including shielding against pathogenic microorganisms and ultraviolet (UV) radiation, regulating body temperature, preventing excessive moisture loss, and coordinating immune responses (1, 2). Skin aging can be categorized into intrinsic (chronological) and extrinsic types. Genetic factors predominantly govern intrinsic aging, whereas extrinsic aging is driven by environmental exposures such as UV radiation and pollution (3). Common features of skin aging include wrinkle formation, dryness, loss of elasticity, laxity, rough texture, and irregular pigmentation.

A pathological hallmark of skin aging is the progressive accumulation of oxidative damage in skin tissue caused by reactive oxygen species (ROS). UV exposure accounts for roughly 80% of skin aging by substantially elevating ROS production in dermal fibroblasts (4). Excess ROS imposes oxidative stress on the skin, leading to lipid peroxidation, protein oxidation, and DNA damage, which collectively accelerate cellular senescence (5). In the early stages of oxidative stress, antioxidant enzymes, such as superoxide dismutase (SOD), catalase (CAT), and glutathione peroxidase (GPx) are upregulated in response to mitigate ROS-mediated damages. However, their capacity to neutralize ROS declines over time, gradually compromising cellular defense mechanisms (6). The resultant oxidative imbalance increases the production of pro-inflammatory mediators, such as C-reactive protein (CRP), interleukin-6 (IL-6), and tumor necrosis factor-alpha (TNF-α) (7, 8). The exacerbated inflammation further contributes to the vicious cycle of oxidative stress, as inflammation and oxidative stress are closely related (9).

Excessive ROS also activates matrix metalloproteinases (MMPs), particularly MMP-9, *via* the mitogen-activated protein kinase (MAPK)-dependent signaling cascade, exacerbating tissue damage (10). Overactivation of MMPs results in the degradation of essential extracellular matrix (ECM) constituents, including collagen, elastin, and fibronectin, reducing skin elasticity, sagging, and wrinkle formation (11). Moreover, the accumulation of advanced glycation end-products (AGEs), formed through the glycation of proteins by reactive carbonyl species, contributes to additional oxidative stress and ECM damage (12, 13). AGEs instigate inflammatory cascades by binding to their receptors on skin cells, contributing to “skin inflammation” (14).

Several clinical studies have demonstrated the photoprotective and skin-enhancing effects of dietary polyphenols. For example, supplementation with green tea polyphenols for 12 weeks significantly reduced UV-induced erythema and improved skin elasticity, roughness, scaling, hydration, and transepidermal water loss (TEWL) in healthy women (15). Similarly, a study by Chiu et al. (16) demonstrated that green tea polyphenols significantly enhanced skin elasticity and reduced erythema, wrinkles, and oxidative stress in participants over 60. Additionally, daily consumption of high-flavanol cocoa powder (326 mg/day) for 12 weeks reduced UV-induced erythema and TEWL while improving skin hydration, roughness, and scaling in female participants (17).

Orange juice is valued for its flavanones, including hesperidin, narirutin, and didymin (18). Most research on the skin benefits of flavanones remains confined to animal models. For example, oral administration of 0.1 mL of water containing 100 mg/kg hesperidin for 5 days a week over 12 weeks significantly inhibited UVB-induced high TEWL, skin thickening, wrinkle formation, collagen fiber loss, and the expression of MMP-9 and pro-inflammatory cytokines (IL-8 and TNF-α) in UVB-irradiated mice (19). Similarly, in UVB-exposed hairless mice, the administration of a citrus-based juice mixture (300 mg/kg) containing narirutin, hesperidin, and ascorbic acid for 10 weeks prevented collagen degradation, and the formation of long and deep wrinkles compared to UVB-treated controls (20). One clinical study reported that consuming red orange extract containing approximately 8.5–9.5 mg/day of flavanones (hesperidin and narirutin) for 56 days improved the skin's reaction to UV exposure. This improvement was evidenced by increased skin antioxidant capacity, skin moisturization, skin elasticity, skin radiance, decreased TEWL, the intensity of melanin staining inside dark spots, wrinkle depth, and UVA-induced lipid peroxidation (21).

Flavanones have shown potential benefits for skin health; however, clinical evidence assessing orange juice, the most accessible flavanone source, on skin-related outcomes remains limited. Therefore, this study investigated whether daily consumption of 100% orange juice attenuates UVB-induced erythema as the primary outcome. Given that UV radiation accelerates photoaging by increasing TEWL, reducing hydration and elasticity, and promoting wrinkle formation, these parameters were assessed on facial and forearm skin as secondary outcomes. In addition, blood biomarkers including SOD, GPx, MMP-9, AGEs, IL-6, TNF-α, and hs-CRP were evaluated as secondary outcomes, as flavanones possess antioxidant and anti-inflammatory properties, and these intrinsic factors are known to influence skin health. This study utilized a randomized, single-blinded, crossover design involving healthy women aged 40–65 to compare the effects of 100% orange juice to an orange-flavored control beverage containing lower flavanone content on skin health.

## 2 Methods

### 2.1 Experimental beverages

Not-From-Concentrate pulp-free orange juice of a national brand was sourced from the Publix grocery store chain (Lakeland, FL, USA). The control beverage was prepared by adding 4.95 g of sucrose to 100 mL of SunnyD (Sunny Delight Beverages Co., Cincinnati, OH, USA), an orange-flavored beverage containing approximately 5% juice concentrate. The control beverage had a taste, mouthfeel, and appearance comparable to orange juice. These two experimental beverages were freshly packed in 12-oz bottles and stored at 4 °C. The nutritional label of orange juice and the control beverage is shown in Table 1. This was a single-blinded trial, as the participants were blinded to the identity of their beverages.

**Table 1:** Nutritional and chemical compositions of orange juice and control beverage per 100 mL.

	**Control beverage^a^**	**Orange juice**
Calories	41	46
Total fat, g	0	0
Saturated fat, g	0	0
Trans fat, g	0	0
Total carbohydrate, g	12	11
Dietary fiber, g	0	0
Total sugars, g	11	10
Added sugars, g	9	0
Protein, g	0	1
Vitamin D, mg	0	0
Vitamin C, mg	38	34
Calcium, microgram	0	8
Iron, mg	0	0.13
Potassium, mg	0	190
Folate, microgram	0	17
Thamin, mg	0.08	0
Hesperidin, mg^b^	5.99 ± 0.23	15.11 ± 0.39^*^
Narirutin, mg^b^	1.67 ± 0.17	6.11 ± 0.10^*^
Didymin, mg^b^	0.68 ± 0.02	1.73 ± 0.02^*^
Total flavanone, mg^b^	8.34 ± 0.38	22.95 ± 0.37^*^

^a^Control beverage was formulated by adding 4.95 g of sucrose to 100 mL of SunnyD orange-flavored beverage. ^b^Values are presented as means ± SEM (*n* = 3 samples) based on HPLC analysis. All other nutritional values were from the product nutrition labels. ^*^Indicates significant differences between orange juice and control beverage (*p* ≤ 0.05, unpaired *t-*test).

### 2.2 Quantification of flavanones in orange juice and control beverage

HPLC-grade methanol and formic acid were purchased from Fisher Scientific (Waltham, MA, USA). Hesperidin, narirutin, and didymin were obtained from TCI Fine Chemicals (Tokyo, Japan), Indofine Chemical Company (Hillsborough, NJ, USA), and MedChemExpress, respectively. Prior to HPLC analysis, all samples were diluted with 100% methanol and filtered using membrane filters with a 0.45 μm pore size (Thermo Fisher Scientific, Waltham, MA, USA).

Flavanones in orange juice and control beverage were quantified using a Thermos Fisher Scientific Vanquish Core HPLC system (Waltham, MA, USA), consisting of a binary pump, autosampler, column compartment, and diode array detector. These compounds were separated on an Agilent ZORBAX SB-C18 column (4.6 × 250 mm; 5 μm). Hesperidin, narirutin, and didymin were eluted using a mobile phase composed of 0.5% formic acid in water (A) and acetonitrile (B) at a flow rate of 0.8 mL/min. The sample injection volume was 20 μL. The gradient was programmed as follows: 0–1 min, 20% B; 1–16 min, 30% B; 16–24 min, 70% B; and 24.1–29 min, 20% B. Detection wavelength was set at 280 nm. HPLC chromatograms are presented in Supplementary Figure S1.

### 2.3 Daily intake of flavanones

As shown in Table 1, the calorie content of the 100% orange juice and the control beverage was comparable, but their flavanone content differed significantly. The orange juice contained 22.95 ± 0.37 mg/100 mL of total flavanones, consistent with previous reports (22–24). The control beverage, formulated with 5% juice concentrate, contained 8.34 ± 0.38 mg/100 mL of flavanones. Although juice concentrate typically contains higher flavanone levels (23), the overall flavanone content in the control beverage remained low due to the small amount of concentrate used. This level was also comparable to a low-flavanone beverage used in a previous trial (25). Based on daily consumption volume (12 oz), participants in the orange juice group received an estimated 81.46 ± 1.34 mg/day of flavanones, while the control beverage group received approximately 29.60 ± 1.32 mg/day, resulting in a differential intake of 51.86 mg/day.

### 2.4 Study design

This randomized, single-blinded, crossover intervention study was conducted at the University of Florida and received approval from the Internal Review Board of the University of Florida (IRB 202100814). The study was registered with Clinicaltrials.gov (NCT04861623). All participants provided informed consent before participation. This was a single-blind design because only participants were blinded to treatments.

**Figure 1:**
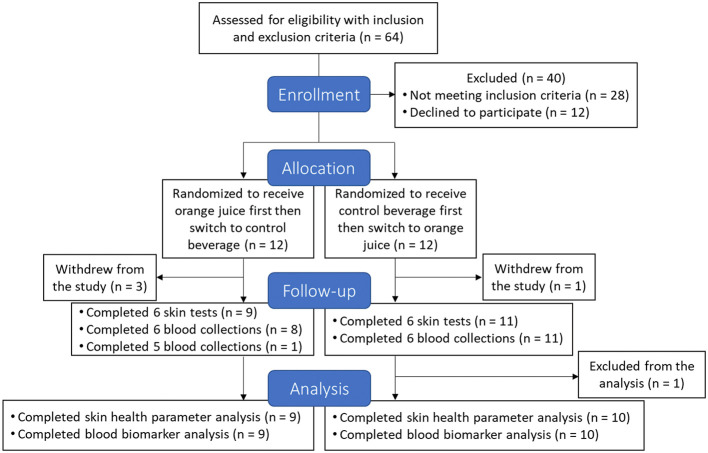
CONSORT flow diagram of the study.

Figure 1 illustrates the CONSORT flow diagram for this study. A total of 24 healthy females (aged 40–65 years) were enrolled with the following inclusion criteria: a BMI between 18.5 and 29.9 kg/m^2^ and skin Fitzpatrick skin types II and III, which are characterized by fair to light olive skin that burns easily and tans minimally with sun exposure. These skin types are commonly used in UVB-induced erythema research due to their consistent and measurable erythema responses. Exclusion criteria included smoking, frequent alcohol consumption, pregnancy, breastfeeding, a history of skin cancer or other clinically significant disorders, and the use of medications that could influence study outcomes. Participants were also excluded if they engaged in sunbathing or tanning bed use, or if they routinely took vitamin, mineral, probiotic, or prebiotic supplements. Throughout the study, participants were instructed to maintain a stable body weight and habitual exercise routine. They were additionally required to avoid all citrus fruits and juices and limit the consumption of probiotic-containing foods to a maximum of one 8 oz serving per day.

One week before the start of the study, the minimal erythema dose (MED) was determined according to the method described by Heckman et al. (26). Briefly, a Daavlin test patch (SCOTT Medical, UK), each containing six exposure windows, was affixed to the participant's back. A SolRx 100-Series handheld UVB-Narrowband phototherapy device (Solarc Systems, Canada) then administered UVB light over varying exposure times (20–70 seconds) on the six windows. After 24 h, participants returned to the laboratory, where a skin colorimeter (CL 440; CK Electronic GmbH, Germany) measured skin color at each exposure site. The a^*^ value, corresponding to the red/green axis, was used to quantify erythema. An increase of approximately 2.5 in a^*^ values between exposed and unexposed skin was used to define the MED.

Randomization was completed prior to the start of the study visits. Participants were recruited on a rolling basis and assigned participant codes in order of enrollment. Permuted block randomization was used, with a block size of four. Within each block, participant codes were randomized using the RAND() function in Microsoft Excel. The two participants with the highest random numbers were assigned to the control beverage in the first session, and the two with the lowest numbers received orange juice. All participants crossed over to the alternate beverage after the washout period. Participant enrollment, randomization, and intervention assignment were performed by the first author.

The study began with a 2-week run-in period where participants start to follow dietary restrictions to adjust their eating habits and reduce potential baseline confounding. Next, 24 participants were randomly assigned to either the orange juice group (*n* = 12) or the control beverage group (*n* = 12) using a permuted block randomization design with a block size of 4. Each participant consumed one 12 oz bottle (355 mL) of the assigned beverage daily for 10 weeks. This phase was followed by a 4-week washout period in which participants continued the dietary restrictions but did not receive any intervention beverages, and no skin health assessments were performed. The washout was intended to minimize potential carryover effects before participants crossed over to the alternate beverage. Prior to each intervention phase, participants confirmed non-pregnancy *via* a urine hCG test (Fisher Scientific, Waltham, MA, USA).

At each study visit, participants were instructed to avoid using any skincare products other than soap for 24 h. Upon arrival, they acclimated in a controlled environment (22.5 ± 0.5 °C, 66 ± 10% relative humidity) for 10 min. Participants then completed a sun exposure and skincare assessment and a food frequency questionnaire (FFQ) (27) to ensure adherence throughout the study. After blood was collected, skin health parameters were measured. A final questionnaire was administered at the end of the study to document any days participants did not consume the assigned juice.

### 2.5 Outcome assessment

#### 2.5.1 Primary outcome: UVB-induced erythema

At each study visit, 2 × MED was administered to participants' backs using a SolRx 100-Series Handheld UVB-Narrowband phototherapy device (Solarc Systems, Canada). Twenty four hours later, participants revisited the lab, and skin color was measured with a skin colorimeter.

#### 2.5.2 Secondary outcome: skin health parameters

Various CK Electronic GmbH (Germany) probes were used to assess skin health parameters. Skin TEWL was determined using a Tewameter TM300, and skin hydration was measured with a Corneometer CM825. Skin pH was evaluated with a Skin-pH-Meter PH 905. Melanin and erythema indices were further analyzed with a Mexameter MX 18. Skin elasticity was assessed with a Cutometer MPA 580 equipped with a 2 mm measuring probe. Each measurement consisted of a 3-second suction at a constant negative pressure of 450 mbar, followed by a 3-second relaxation period. Four parameters were subsequently derived: gross elasticity (Ua/Uf), net elasticity (Ur/Ue), viscoelasticity (Uv/Ue), and biological elasticity (Ur/Uf). In addition, a Visioscan VC 20plus camera provided measurements of skin surface characteristics, including smoothness, roughness, scaliness, and wrinkles. All measurements were performed in triplicate and collected at the face (between the nose and ear, over the cheekbone area) and on the inner forearm (midway between the wrist and elbow joint).

#### 2.5.3 Secondary outcome: biomarkers of oxidative stress and inflammation

Blood samples (2 tubes, 20 mL total) were collected at each study visit using K2 EDTA-coated tubes (BD Vacutainer, BD, Franklin Lakes, NJ). Plasma was separated by centrifugation at 1,500 rpm for 15 min at 4 °C. The remaining blood was rinsed with isotonic saline and then centrifuged to isolate erythrocytes for antioxidant enzyme analyses. All samples were stored at −80 °C until further analysis.

SOD and GPx activities were measured in erythrocytes using colorimetric assay kits (Caymen Chemicals, Ann Arbor, MI, USA). Advanced glycation end products (AGEs) were quantified via a competitive ELISA kit (Cell Biolabs, Inc., San Diego, CA, USA). Plasma levels of TNF-α, IL-6, metalloproteinase-9 (MMP-9), and high-sensitivity C-reactive protein (Hs-CRP) were determined using ELISA kits (Invitrogen, Waltham, MA, USA). All assays were performed according to the manufacturer's instructions, and absorbance was measured with a microplate reader (BioTek Instruments, Inc., Winooski, VT, USA).

### 2.6 Sample size and statistical analysis

To detect between-group differences in UVB-induced erythema (the primary outcome of this study), using an effect size of 1.5 from published data, a sample size of 18 participants was estimated to be sufficient for a 2-treatment crossover design, with 80% power at a 5% significance level (two-tailed). To ensure adequate power while accounting for a potential dropout rate, 24 participants were enrolled. Bar graphs were generated using GraphPad Prism version 10.4.1 (GraphPad Software, San Diego, CA, USA). All statistical analyses were performed using RStudio (R Foundation, Vienna, Austria). UVB-induced erythema, skin health parameters, and biomarkers were analyzed using mixed-model analyses of covariance (ANCOVA). The model included baseline as a covariate, treatment (orange juice vs. control beverage), and sequence (the order of treatments) as fixed effects, with participants as random effects. An autoregressive correlation structure was specified to model within-participant correlations over time. A treatment × sequence interaction was included to detect potential carryover effects. Model diagnostics included examining residual plots, histograms, and Q–Q plots to assess normality. Logarithmic transformations were applied to response variables if deviations from normality were observed. Statistical significance was defined as *p* ≤ 0.05.

## 3 Results

### 3.1 Characteristics of subjects at baseline

A total of 24 participants were initially enrolled and randomized into their respective treatment groups. However, four individuals withdrew before completing the first session due to scheduling conflicts, health concerns, or relocation. Additionally, one participant consumed the beverage for only 9 weeks in each session. As a result, 19 participants completed both 10-week beverage consumption sessions, and their skin health and biomarker data were included in the final analysis. These participants self-reported a total of 22 days of not drinking the assigned beverage on the final questionnaire, corresponding to a 99% adherence rate. Baseline characteristics, including age, weight, BMI, Fitzpatrick skin type, and MED, are presented in Table 2. A significant age difference (*p* = 0.0029) was observed during the first session, primarily due to recruitment challenges and the concurrent timing of recruitment and study implementation, which limited control over age matching. Supplementary Table S1 summarizes FFQ data from each study visit, confirming that participants maintained consistent dietary habits throughout the study.

**Table 2:** Baseline characteristics during the first juice drinking session.

	**Control beverage (*n =* 10)**	**Orange juice (*n =* 9)**	***p*-value**
Age (yr)	55.20 ± 2.12	46.33 ± 1.72	0.0048^*^
Weight (lb)	150.4 ± 8.1	156.1 ± 7.9	0.62
BMI (kg/m^2^)	26.07 ± 1.21	26.49 ± 0.94	0.79
Fitzpatrick skin type	2.90 ± 0.23	2.78 ± 0.15	0.66
MED (J/cm^2^)	1.83 ± 0.18	1.43 ± 0.19	0.14

Results are expressed as means ± SEM. Participants were initially assigned to one juice in the first session and switched to another in the second session. The *p* values were calculated using an unpaired *t-*test with Welch's correction. ^*^Significant difference between groups.

### 3.2 Orange juice consumption did not mitigate UVB-induced erythema

The difference in skin redness (Δa^*^) measured before and after UVB irradiation was not significantly affected by orange juice consumption (Table 3).

**Table 3:** Changes in skin redness (Δa^*^) induced by UVB light exposure of two minimal erythema doses.

	**Time**	**Control beverage**	**Orange juice**	***p*-value**
Δa^*^	Baseline	8.56 ± 0.75	8.30 ± 0.82	
	Week 5	8.34 ± 0.77	8.10 ± 0.84	0.86
	Week 10	8.07 ± 0.84	8.44 ± 0.95	0.85

Results are expressed as means ± SEM (*n* = 19). Δa^*^ values were obtained from triplicate measurements at each time point. The *p* values between orange juice and control beverages are from ANCOVA analyses.

### 3.3 Orange juice consumption had marginal effects on skin health

Ten weeks of orange juice significantly decreased wrinkles on the inner forearm (*p* = 0.0480) but not after 5 weeks. A non-significant trend of lower roughness was observed in the orange juice group compared to the control beverage group at week 10 (*p* = 0.06). Additionally, 10 weeks of orange juice consumption improved facial hydration (*p* = 0.0499), while the opposite was observed after 5 weeks (*p* = 0.02) (Table 4). Facial skin pH significantly decreased after 5 weeks of orange juice consumption compared to the control beverage (*p* = 0.01), but this effect was not observed at week 10. All other skin health parameters remained unchanged.

**Table 4:** Facial skin health parameters at baseline, week 5, and week 10 in women consuming a control beverage or orange juice.

**Skin health parameters**	**Time**	**Control beverage**	**Orange juice**	***p*-value**
Transepidermal water loss (g/h/m^2^)	Baseline	11.98 ± 1.16	10.90 ± 0.94	
	Week 5	10.24 ± 0.99	11.10 ± 0.86	0.10
	Week 10	11.50 ± 1.19	10.75 ± 0.62	0.89
Hydration	Baseline	54.52 ± 2.64	60.51 ± 3.37	
	Week 5	60.94 ± 3.17	55.47 ± 3.02	0.02^*^
	Week 10	57.81 ± 2.96	66.86 ± 3.18	0.05^*^
Gross elasticity (%)	Baseline	67.51 ± 3.80	60.12 ± 3.47	
	Week 5	64.05 ± 3.12	66.05 ± 3.19	0.15
	Week 10	61.03 ± 2.47	64.28 ± 2.71	0.26
Net elasticity (%)	Baseline	54.11 ± 3.36	48.86 ± 3.0	
	Week 5^a^	53.86 ± 2.56	57.33 ± 3.26	0.10
	Week 10	51.22 ± 2.25	54.93 ± 2.62	0.10
Viscoelasticity	Baseline	47.59 ± 3.63	49.26 ± 3.79	
	Week 5	52.92 ± 3.25	54.26 ± 4.11	0.99
	Week 10	53.71 ± 2.61	54.46 ± 4.11	0.99
Biological elasticity (%)	Baseline	37.09 ± 2.45	33.22 ± 2.17	
	Week 5	35.64 ± 2.05	37.55 ± 2.34	0.11
	Week 10	33.38 ± 1.28	35.86 ± 1.88	0.08
Wrinkle	Baseline	125.0 ± 9.5	131.5 ± 10.7	
	Week 5	151.2 ± 15.6	159.7 ± 19.2	0.79
	Week 10	156.5 ± 22.9	127.3 ± 9.6	0.20
Smoothness	Baseline	339.0 ± 17.3	356.2 ± 23.8	
	Week 5	373.3 ± 27.9	375.4 ± 31.9	0.68
	Week 10	359.7 ± 37.4	322.2 ± 18.5	0.28
Roughness	Baseline	3.14 ± 0.31	3.33 ± 0.26	
	Week 5^a^	3.56 ± 0.31	3.47 ± 0.35	0.38
	Week 10	2.91 ± 0.17	3.22 ± 0.18	0.30
Scaliness	Baseline	0.26 ± 0.08	0.30 ± 0.10	
	Week 5	0.21 ± 0.05	0.22 ± 0.07	0.78
	Week 10	0.34 ± 0.11	0.23 ± 0.05	0.13
pH	Baseline	6.06 ± 0.06	6.11 ± 0.04	
	Week 5	6.32 ± 0.05	6.14 ± 0.05	0.01^*^
	Week 10	6.19 ± 0.06	6.14 ± 0.07	0.54
Melanin index	Baseline	92.18 ± 10.17	95.39 ± 8.36	
	Week 5^a^	94.51 ± 10.07	91.04 ± 8.71	0.22
	Week 10^a^	98.26 ± 9.63	94.67 ± 10.07	0.18
Erythema index	Baseline	205.4 ± 19.3	200.4 ± 17.0	
	Week 5^a^	202.2 ± 18.0	202.3 ± 14.1	0.19
	Week 10^a^	199.0 ± 17.9	202.5 ± 16.1	0.48

Results are expressed as means ± SEM (*n* = 19). All skin health parameters were measured in triplicate at each time point. ^a^Data were log-transformed. The *p* values between control beverages and orange juice are from ANCOVA analyses. ^*^Significant difference between control beverage and orange juice.

**Table 5:** Inner forearm skin health parameters at baseline, week 5, and week 10 in women consuming a control beverage or orange juice.

**Skin health parameters**	**Time**	**Control beverage**	**Orange juice**	***p*-Value**
Transepidermal water loss (g/h/m^2^)	Baseline	8.44 ± 0.97	8.70 ± 0.87	
	Week 5^a^	8.20 ± 0.88	7.92 ± 0.73	0.98
	Week 10^a^	8.69 ± 0.94	9.29 ± 1.18	0.84
Hydration	Baseline	38.70 ± 2.33	39.37 ± 2.66	
	Week 5	41.57 ± 2.34	42.67 ± 2.24	0.85
	Week 10^a^	46.40 ± 2.13	49.32 ± 4.22	0.85
Gross elasticity (%)	Baseline	74.01 ± 2.48	76.00 ± 2.37	
	Week 5	71.59 ± 1.72	71.98 ± 2.13	0.87
	Week 10	73.44 ± 1.94	75.84 ± 2.68	0.51
Net elasticity (%)	Baseline	76.79 ± 4.68	75.98 ± 3.16	
	Week 5^a^	73.10 ± 3.58	72.01 ± 2.90	0.95
	Week 10	78.34 ± 3.51	80.74 ± 3.91	0.40
Viscoelasticity	Baseline	47.10 ± 3.03	45.42 ± 2.98	
	Week 5	49.25 ± 2.40	45.66 ± 2.55	0.37
	Week 10	51.84 ± 2.03	51.89 ± 2.69	0.79
Biological elasticity (%)	Baseline	51.96 ± 2.76	52.71 ± 2.32	
	Week 5	48.87 ± 2.10	49.73 ± 2.15	0.72
	Week 10	51.75 ± 2.29	53.63 ± 2.89	0.54
Wrinkle	Baseline	86.51 ± 5.77	80.54 ± 4.88	
	Week 5	83.68 ± 4.99	81.46 ± 4.73	0.74
	Week 10	94.07 ± 5.22	80.00 ± 3.58	0.05^*^
Smoothness	Baseline	242.1 ± 11.1	229.3 ± 9.37	
	Week 5	240.3 ± 9.6	231.0 ± 7.15	0.73
	Week 10	261.1 ± 11.3	238.6 ± 8.01	0.10
Roughness	Baseline	2.11 ± 0.12	2.20 ± 0.15	
	Week 5	2.33 ± 0.17	2.16 ± 0.17	0.74
	Week 10	2.38 ± 0.15	2.05 ± 0.13	0.06
Scaliness	Baseline	0.17 ± 0.06	0.16 ± 0.05	
	Week 5	0.16 ± 0.05	0.12 ± 0.06	0.37
	Week 10	0.18 ± 0.06	0.16 ± 0.05	0.72
pH	Baseline	6.06 ± 0.07	5.84 ± 0.06	
	Week 5	6.20 ± 0.06	6.07 ± 0.05	0.60
	Week 10	6.07 ± 0.05	5.97 ± 0.07	0.37
Melanin index	Baseline	63.68 ± 9.69	65.59 ± 10.71	
	Week 5	62.97 ± 9.57	67.65 ± 11.44	0.35
	Week 10	64.70 ± 10.54	68.60 ± 11.16	0.69
Erythema index	Baseline	152.1 ± 15.6	160.1 ± 15.2	
	Week 5	154.8 ± 15.2	152.5 ± 11.5	0.59
	Week 10	152.0 ± 13.1	168.7 ± 14.3	0.30

Results are expressed as means ± SEM (*n* = 19). All skin health parameters were measured in triplicate at each time point. ^a^Data were log-transformed. The *p* values between control beverage and orange juice are from ANCOVA analyses. ^*^Significant difference between control beverage and orange juice.

### 3.4 Orange juice consumption did not affect oxidative stress or inflammation biomarkers

**Figure 2:**
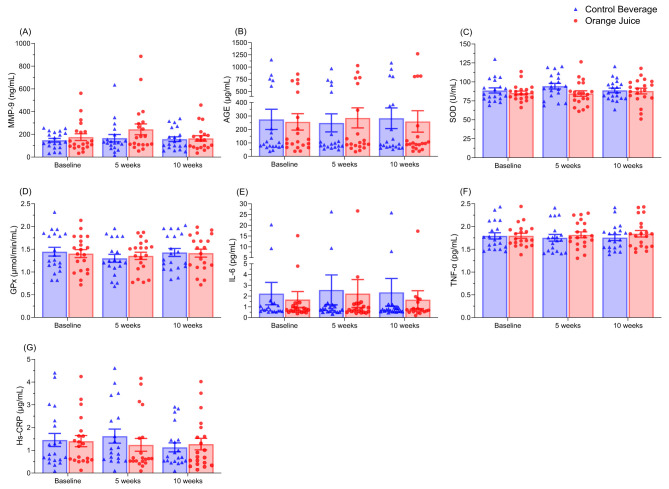
The orange juice did not affect plasma levels of MMP-9 **(A)**, AGE **(B)**, SOD **(C)**, GPx **(D)**, IL-6 **(E)**, TNF-α **(F)**, or Hs-CRP **(G)** compared to the control beverage. Results are expressed as means ± SEM (*n* = 19). MMP-9, matrix metalloproteinase-9; AGE, advanced glycation end-products; SOD, superoxide dismutase; GPx, glutathione peroxidase; IL-6, interleukin-6; TNF-α, tumor necrosis factor-α; Hs-CRP, high-sensitivity C-reactive protein.

Blood levels of MMP-9, AGE, SOD, GPx, IL-6, TNF-α, or Hs-CRP were not affected by orange juice consumption after 5 or 10 weeks (Figure 2).

## 4 Discussion

In the present study, we investigated the effects of 100% orange juice consumption on UVB-induced erythema as the primary outcome, with additional skin health parameters related to photoaging and biomarkers of oxidative stress and inflammation assessed as secondary outcomes. Using a randomized, single-blinded, crossover design, we compared 100% orange juice with an orange-flavored control beverage in healthy women aged 40–65. Flavanones, the primary bioactive polyphenols in orange juice, have demonstrated protective effects against photoaging in animal models and are well recognized for their antioxidant and anti-inflammatory properties. Although the daily flavanone intake was significantly higher in the orange juice group compared to the control beverage group, this difference did not result in measurable improvements in skin health.

Ten weeks of orange juice consumption did not attenuate UVB-induced erythema on participants' lower backs, indicating no observable protective effects against UVB-mediated skin damage. The handheld UVB-narrowband phototherapy device used in this study induced localized erythema resembling mild sunburn, a well-established indicator of acute UV-induced skin injury. The lower back was selected as the test site due to its typically low sun exposure and high sensitivity to controlled UVB irradiation. Moreover, UVB-induced erythema was chosen as the primary outcome in this study due to its relevance as a clinical marker of photoaging and its potential to reflect acute UV-induced skin damage. Notably, a clinical trial reported that 56 days of supplementation with a red-orange extract complex containing 2.8–3.2 mg of anthocyanins (cyanidin-3-glucoside) and 8.5–9.5 mg of flavanones (hesperidin and narirutin) significantly reduced UV-induced erythema (21). In the present study, although the orange juice provided a higher daily flavanone content (81.46 mg/day) over the 70-day intervention period, no reduction in UVB-induced erythema was observed. One possible explanation for this discrepancy is the absence of anthocyanins in orange juice, which may contribute to photoprotective effects. Importantly, relying solely on UVB-induced erythema as a marker may not fully capture the extent of UVB-induced skin damage, as UVB radiation also induces keratinocyte apoptosis, epidermal thickening, and dermal inflammation (43). Although erythema serves as a valuable acute marker, it may not reflect subclinical or molecular-level changes. These findings highlight the need for additional outcome measures to better assess the protective effects of orange juice against UVB-induced skin damage.

Facial and forearm skin are more susceptible to photoaging due to frequent and prolonged exposure to UV radiation. In addition to UV exposure, intrinsic factors such as hydration status and hormonal levels also contribute to skin health. Insufficient water intake has been identified as an important factor affecting skin hydration (28). According to a CDC survey, 43% of U.S. adults reported consuming only 0–3 cups of water per day, a level considered inadequate (29). In this study, facial hydration was assessed in the cheekbone area between the nose and ear. A study has shown that this region typically exhibits lower hydration compared to other areas such as the volar forearm, neck, forehead, periauricular area, and chin (30). As shown in Table 4, facial hydration was significantly higher in the control beverage group at week 5, but higher in the orange juice group at week 10. This fluctuation may reflect individual variability in overall fluid intake across the study period.

A Visioscan VC 20plus camera captures detailed images of skin topography, including wrinkles, smoothness, roughness, and scaliness, using UVA light that penetrates skin layers. This allows for a comprehensive evaluation of skin surface texture and health. Specifically, the camera provides indices quantifying wrinkle quantity and depth, allowing visualization of fine lines and pores, features potentially influenced by localized hair and skin hydration. Additionally, indices measuring surface roughness or unevenness, often affected by environmental conditions, reflect textural irregularities important for evaluating skin health. Ten weeks of orange juice consumption significantly reduced wrinkle formation on the inner forearm and showed a trend toward decreased roughness. Decreased wrinkles are typically associated with reduced skin roughness. Although the *p*-value of roughness (*p* = 0.06) does not meet the conventional threshold for statistical difference, the effect size (Cohen's *d* = 0.70) suggests a moderate-to-large difference between the groups, indicating that further investigation with a larger sample size may be warranted. No significant differences were observed in skin smoothness or scaliness between the two treatment groups, which may be explained by the lack of changes in skin hydration, as these parameters are closely related. Additionally, no changes in skin topography were detected on the facial skin. These findings suggest that while the effects of orange juice consumption on skin surface topography were limited, the observed improvement in forearm wrinkles and the trend of toward reduced roughness indicate potential benefits.

While skin pH was not a primary focus among the secondary outcomes monitored in this study, maintaining a slightly acidic pH is considered a hallmark of healthy skin, as it plays a critical role in barrier function and antimicrobial defense (31). Skin pH can be influenced by several endogenous factors, such as sebum production, hydration levels, sweat, and the skin microbiota (32). As shown in Table 4, facial pH was significantly higher in the control beverage group than in the orange juice group at week 5; however, no significant difference was observed at week 10. The overall stability of skin pH throughout the study suggests that participants maintained a healthy skin condition and were not experiencing any signs of infection.

Oxidative stress arises from an imbalance between ROS and antioxidant defenses. Alterations in the cooperative function of antioxidant enzymes can disrupt the equilibrium between ROS production and detoxification (33). Elevated levels of SOD and GPx, two key enzymes involved in ROS neutralization, are often indicative of increased oxidative stress and heightened ROS activity. Moreover, elevated ROS levels are linked to inflammation, characterized by increases in IL-6, TNF-α, and CRP. Citrus flavanones are recognized for their antioxidant and anti-inflammatory properties (34, 35). However, in the present study, orange juice consumption providing approximately 81.46 mg/day of flavanones over 10 weeks did not result in significant changes in erythrocyte SOD or GPx activities, nor in plasma IL-6 or TNF-α concentrations.

Photoaging can contribute to ROS-mediated damage by upregulating MMP-9 in response to high ROS levels, which promotes collagen degradation and subsequent wrinkle formation (36). AGEs also play a major role in skin pathophysiology by influencing cellular homeostasis, promoting collagen cross-linking, triggering inflammation and oxidative stress, and impairing the skin barrier. These processes collectively reduce elasticity, exacerbate wrinkles, lead to uneven pigmentation, hinder wound healing, and increase susceptibility to external damage (37). Numerous studies have employed UVB irradiation in mouse models to induce inflammation and oxidative damage, thereby evaluating photodamage and the photoprotective effects of various interventions (38–41). In this study, UVB exposure induced localized erythema on participants' backs but did not alter plasma biomarkers of oxidative stress or inflammation. These findings suggest that participants were otherwise healthy and did not experience oxidative or inflammatory damage during the study period. Therefore, participants' oxidative and inflammatory status was likely driven by intrinsic factors rather than the acute UVB exposure applied in this trial.

In this study, participants consumed orange juice providing 81.46 mg/day of flavanones. In comparison, a trial demonstrated that 8 weeks of consuming flavanone-rich orange juice providing 305 mg/day significantly improved cognitive function in older adults compared to a low-flavanone control beverage. Similarly, another study showed that consuming orange juice containing a total of hesperidin and narirutin for 4 weeks led to significant vascular protective effects in healthy overweight men aged 50–65 years compared to a control beverage (42). These previous studies utilized higher flavanone doses than those tested in the current study. Therefore, the lack of observed improvement in skin health outcomes in the present study may be due to the lower flavanone dosage, highlighting a potential requirement for a higher flavanone intake to achieve measurable skin-protective effects.

This trial employed commercially available 100% orange juice and an orange-flavored control beverage in amounts reflective of typical daily consumption, with minimal dietary restrictions, thereby offering a more realistic dietary intervention. A key strength of the study was the repeated assessment of skin health parameters and biomarkers throughout each intervention phase, allowing for the evaluation of longitudinal changes. Additionally, the crossover design enabled participants to serve as their own control, thereby reducing inter-individual variability. Treatment × sequence interaction analyses were performed for all skin health parameters and biomarkers, with no significant carryover effects observed. Given the 10-week intervention period and crossover design conducted in Florida, variability in UV exposure to the facial and forearm skin could have introduced a potential confounding factor. To mitigate this, participants were instructed to avoid prolonged sun exposure and to consistently apply sunscreen throughout the study. Furthermore, no significant changes were observed in melanin and erythema indexes, supporting that UV exposure was effectively controlled. Limitations of this study include the lack of hormonal assessments among female participants aged 40–65, a group likely to be at varying stages of menopause. Hormonal status can influence skin health and may have introduced variability in outcomes. Additionally, daily hydration levels were not monitored, which may also impact skin-related measures. Finally, future studies using orange juice with higher flavanone content may reveal more robust effects on skin health.

## 5 Conclusion

This randomized, single-blinded, crossover trial evaluated the effects of 100% orange juice consumption on skin health. Participants in the orange juice group received significantly higher daily flavanone content compared to those receiving control beverage. Orange juice consumption led to a significant reduction in forearm wrinkles and showed a trend toward reduced skin roughness. However, no significant improvements were observed in UVB-induced erythema, other skin health parameters, or biomarkers of oxidative stress and inflammation. These findings suggest that orange juice consumption had a marginal effect on skin health.

## Data Availability

The original contributions presented in the study are included in the article/Supplementary material, further inquiries can be directed to the corresponding author.
