# Supplementary material for: Ten weeks of 100% orange juice consumption had a marginal effect on women's skin health compared to a low-flavanone orange-flavored control beverage: a pilot randomized trial
Source: Front Nutr. 2025 Sep 3;12:1648394. doi: 10.3389/fnut.2025.1648394 (PMC12442491; doi:10.3389/fnut.2025.1648394)
Supplement: Supplementary file 1 [file Image_1.pdf]

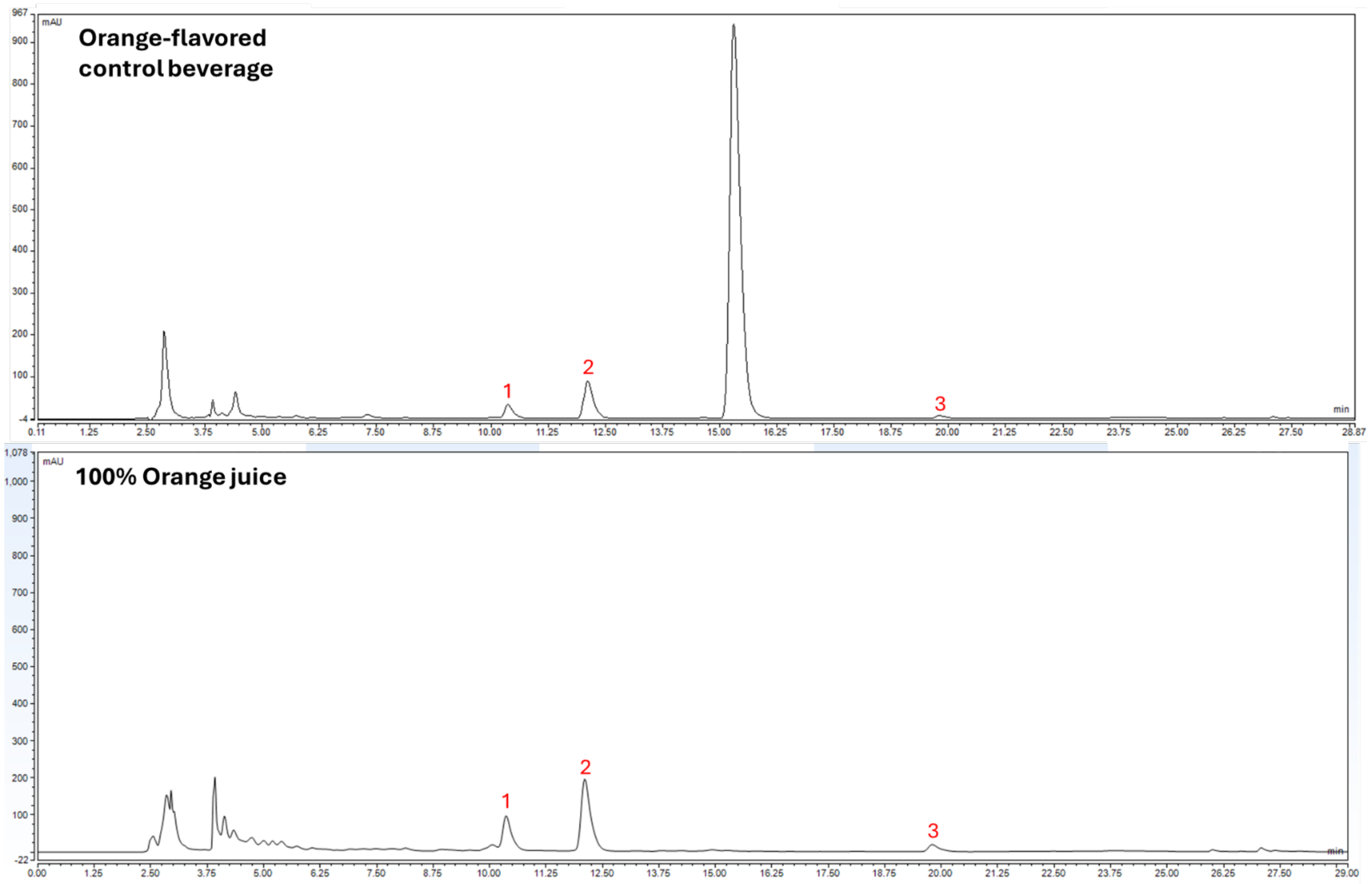

Figure S1. HPLC chromatograms of the orange-flavored control beverage and 100% orange juice. Peaks 1–3 correspond to narirutin, hesperidin, and didymin, respectively.
