# Supplementary material for: Ten weeks of 100% orange juice consumption had a marginal effect on women's skin health compared to a low-flavanone orange-flavored control beverage: a pilot randomized trial
Source: Front Nutr. 2025 Sep 3;12:1648394. doi: 10.3389/fnut.2025.1648394 (PMC12442491; doi:10.3389/fnut.2025.1648394)
Supplement: Supplementary file 2 [file Table_1.docx]

Table S1. Self-reported food frequency at baseline, week 5, and week 10 during the intervention period.

| Foods | Time | Control beverage | Orange juice |
| --- | --- | --- | --- |
| Bread^a^ | Baseline | 1.53 ± 0.16 | 1.68 ± 0.19 |
|  | Week 5 | 1.63 ± 0.16 | 1.63 ± 0.14 |
|  | Week 10 | 1.74 ± 0.17 | 1.47 ± 0.14 |
| Vegetables^a^ | Baseline | 2.53 ± 0.14 | 2.63 ± 0.14 |
|  | Week 5 | 2.37 ± 0.16 | 2.42 ± 0.14 |
|  | Week 10 | 2.42 ± 0.14 | 2.47 ± 0.16 |
| Fruit^a^ | Baseline | 2.21 ± 0.18 | 1.89 ± 0.19 |
|  | Week 5 | 2.16 ± 0.21 | 2.05 ± 0.16 |
|  | Week 10 | 2.05 ± 0.18 | 1.89 ± 0.19 |
| Milk or Yogurt^a^ | Baseline | 1.47 ± 0.16 | 1.58 ± 0.14 |
|  | Week 5 | 1.53 ± 0.14 | 1.53 ± 0.14 |
|  | Week 10 | 1.63 ± 0.19 | 1.63 ± 0.18 |
| Rice or Pasta^a^ | Baseline | 1.58 ± 0.16 | 1.53 ± 0.12 |
|  | Week 5 | 1.47 ± 0.12 | 1.47 ± 0.14 |
|  | Week 10 | 1.37 ± 0.11 | 1.42 ± 0.12 |
| Vegetable oils^a^ | Baseline | 1.68 ± 0.17 | 1.55 ± 0.14 |
|  | Week 5 | 1.47 ± 0.14 | 1.58 ± 0.14 |
|  | Week 10 | 1.58 ± 0.16 | 1.68 ± 0.15 |
| Alcoholic beverages^a^ | Baseline | 1.05 ± 0.05 | 1.05 ± 0.05 |
|  | Week 5 | 1.05 ± 0.05 | 1.00 ± 0.00 |
|  | Week 10 | 1.00 ± 0.00 | 1.11 ± 0.07 |
| Breakfast cereals^a^ | Baseline | 1.11 ± 0.07 | 1.11 ± 0.07 |
|  | Week 5 | 1.16 ± 0.12 | 1.16 ± 0.09 |
|  | Week 10 | 1.21 ± 0.12 | 1.11 ± 0.07 |
| Meat^b^ | Baseline | 1.74 ± 0.19 | 1.68 ± 0.15 |
|  | Week 5 | 1.74 ± 0.19 | 1.68 ± 0.19 |
|  | Week 10 | 1.63 ± 0.18 | 1.68 ± 0.19 |
| Sausages^b^ | Baseline | 1.00 ± 0.00 | 1.00 ± 0.00 |
|  | Week 5 | 1.00 ± 0.00 | 1.00 ± 0.00 |
|  | Week 10 | 1.00 ± 0.00 | 1.00 ± 0.00 |
| Cheese^b^ | Baseline | 1.53 ± 0.14 | 1.58 ± 0.16 |
|  | Week 5 | 1.68 ± 0.15 | 1.68 ± 0.15 |
|  | Week 10 | 1.58 ± 0.16 | 1.68 ± 0.15 |
| Animal fat (butter, lard)^b^ | Baseline | 1.74 ± 0.19 | 1.47 ± 0.16 |
|  | Week 5 | 1.58 ± 0.16 | 1.47 ± 0.16 |
|  | Week 10 | 1.37 ± 0.14 | 1.37 ± 0.16 |
| Vegetable oil^b^ | Baseline | 1.42 ± 0.16 | 1.47 ± 0.12 |
|  | Week 5 | 1.37 ± 0.11 | 1.53 ± 0.16 |
|  | Week 10 | 1.26 ± 0.10 | 1.42 ± 0.16 |
| Fast food^b^ | Baseline | 1.00 ± 0.00 | 1.00 ± 0.00 |
|  | Week 5 | 1.05 ± 0.05 | 1.00 ± 0.00 |
|  | Week 10 | 1.00 ± 0.00 | 1.00 ± 0.00 |
| Fish^c^ | Baseline | 1.32 ± 0.13 | 1.37 ± 0.14 |
|  | Week 5 | 1.32 ± 0.13 | 1.32 ± 0.13 |
|  | Week 10 | 1.26 ± 0.10 | 1.32 ± 0.11 |
| Legumes^c^ | Baseline | 1.58 ± 0.18 | 1.84 ± 0.16 |
|  | Week 5 | 1.58 ± 0.18 | 1.68 ± 0.17 |
|  | Week 10 | 1.68 ± 0.17 | 1.63 ± 0.16 |
| Nuts^c^ | Baseline | 1.84 ± 0.21 | 1.63 ± 0.16 |
|  | Week 5 | 1.79 ± 0.16 | 1.74 ± 0.17 |
|  | Week 10 | 1.47 ± 0.16 | 1.74 ± 0.19 |

Data are expressed as means ± SEM (n = 19). Results are based on a validated self-reported semi-quantitative food frequency questionnaire that participants filled out before and after each study visit. ^a^1: <1 time per day, 2: 1 time per day, 3: ≥2 times per day. ^b^1: <4 times per week, 2: 4-6 times per week, 3: ≥7 times per week. ^c^1: <2 times per day, 2: 2-3 times per day, 3: ≥4 times per day. The variations in food frequency were analyzed using a Friedman test.
